# Remedy for Toothache

**Published:** 1847-05

**Authors:** 


*Remedy for Toothache.*—M. Cottereau recommends, for the alleviation of toothache, an ethereal solution of camphor, containing ammonia. Sulphuric ether is saturated in the cold with camphor, and two or three drops of ammonia are added. In this way an ammoniacal camphorated ether is obtained, which should be kept in a well-stopped bottle. This liquid acts as a cautery to carious teeth, and it immediately relieves toothache. M. Cottereau states that he has employed it for four years, and its application has always been attended with success. The rapid evaporation of the ether causes a slight deposit of the camphor in the dental cavity, and thus protects the nerve from the air. The ammonia has a cauterizing action.—*Lond. Med. Gaz.*

